# The role of attention in the relationship between early life stress and depression

**DOI:** 10.1038/s41598-020-63351-7

**Published:** 2020-04-09

**Authors:** Yu Mao, Hong Xiao, Cody Ding, Jiang Qiu

**Affiliations:** 1 Key laboratory of cognition and personality (SWU), Ministry of Education, Chongqing, 400715 China; 2 Department of psychology, Southwest University, Chongqing, 400715 China; 3 Education science & professional programs, University of Missouri-St. Louis, Louis, United States

**Keywords:** Social neuroscience, Neuroscience

## Abstract

Early life stress (ELS) can be very harmful to an individual’s wellbeing and brain development. It is well established that childhood maltreatment is a significant risk factor for depression. ELS is positively correlated with depressive symptoms both in major depression disorder patients and healthy individuals, but the cognitive and neural mechanisms underlying this association are still unclear. In the present study, we calculate the within/between-network connectivity in 528 college students, and Pearson correlation was performed to investigate the relationship between network measures and ELS. Additionally, the same method was applied to verify these results in another sample. Finally, mediation analysis was performed to explore the cognitive and neural mechanisms regarding the association between ELS and depression. Correlation analysis indicated that ELS was positively correlated with the within-network connectivity of the ventral attention network (VAN), the dorsal attention network (DAN), the salience network (SN), the somatosensory network (SMN) and the between-network connectivity of ventral attention network-dorsal attention network (VAN-DAN), ventral attention network- somatosensory network (VAN-SMN), and ventral attention network-visual network (VAN-VN). Validation results indicated that ELS is associated with the within-network connectivity of VAN and DAN. Mediation analysis revealed that attention bias and the within-network connectivity of VAN could mediated the relationship between ELS and depression. Both behavioral and neural evidence emphasize ELS’s influence on individual’s emotion attention. Furthermore, the present study also provides two possible mediation models to explain the potential mechanisms behind the relationship between ELS and depression.

## Introduction

ELS refers to traumatic life event occurring childhood, including emotional, physical, and sexual abuse, emotional and physical neglect. Evidence suggests that ELS leads to prolonged phases of stress and even predict negative outcomes over the course of a lifetime^1^. In particular, maltreated children are more likely to develop posttraumatic stress disorder (PTSD)^2,3^, major depression disorder (MDD)^4–6^, anxiety disorder^7,8^, and substance abuse^9,10^ in the future. In addition, childhood maltreatment is also associated with lower level of well-being^11,12^, worse educational outcomes^13,14^ and shortened life expectancy^15^. With the development of MRI technology, Empirical researches have explored the influence of ELS on individual’s brain. The most prominent results oriented to the hippocampus and amygdala-brain regions which are sensitive to stress^16–19^. For example, adults who had experienced ELS demonstrated smaller volume of hippocampus, compared with controls^20^. ELS was associated with decreased amygdala volume^21^. Besides, studies have indicated that ELS also influence the volume of the prefrontal cortex, sensorimotor cortex and its related fiber-tract integrity^21^.These findings provide valuable evidences for comprehending how ELS modifies individual’s brain. However, recent studies confirmed that the human brain was consisted with complicated functional networks, and cognitive and affective processing are supported by the interaction of these networks, instead of the function of a single brain area. Thus, new pointcut is needed to explore the modifications and alternations that ELS makes to brain networks.

It has been suggested that identifying and processing emotional visual information is essential for emotion regulation^22^ and emotional attention bias is one of the most significant cognitive features in individuals who were exposed to ELS^23,24^. Empirical studies have supported that individuals with ELS demonstrated attention bias either toward or away from emotional stimuli^9,25–27^. Attention bias arises as early as 150 milliseconds after the presentation of the stimulus and modulate reactions occurring at this time scale usually depend on stimulus-driven (as opposed to voluntary) attention, a function rely on the participation of the VAN^9,28^. Considering the role of VAN in orienting and responding to salient stimuli^29^. It is reasonable to infer that attention bias might depend on the function of VAN.

ELS is believed to be a key risk factor for developing major depression disorder (MDD)^30,31^. Furthermore, previous studies have shown that there is a significant relationship between ELS and depression mood^32,33^. But the neural and cognitive mechanisms behind this association is still ambiguous. Thus, it is of great significance to discover the cognitive mechanism of how childhood maltreatment influence individual’s depressive emotion. In a sense, it may help to propose relevant interventions to decrease the negative influence of ELS. Evidence demonstrated that experimentally inducing attention bias to emotionally valanced stimulus causes measurable changes in mood in healthy individuals^34,35^, whereas attention bias modification can alleviate depressive symptoms^36,37^. Attention bias to traumatic experience might be an adaptive strategy to prepare for the forthcoming threat^38^, but from a long-term perspective, it will interfere with subsequent information processing, which might lead to a consistent negative influence. Presumably, we can infer that individual exposed to ELS might develop attention bias to emotionally valenced, which is detrimental to effective emotion regulation, and ultimately result in high level of depression when facing with stress. Both clinical and empirical studies emphasize the role of attention bias in developing and maintaining depression^39,40^. Thus we hypothesized that attention bias might play a key role in the relationship between ELS and depression,

Thus, the present study was designed to explore the influence of ELS on individual’s brain networks. Furthermore, we also attempted to decipher the neural and cognitive mechanism sustaining the association between ELS and depression. First, we investigate the relationship between ELS and within/between network connectivity with a big sample of 528 college students. To verify the results, machine learning method was applied to another small sample. Second, mediation analysis was performed to test whether there were particular neural basis mediate the relationship between ELS and depression. Finally, we explored whether attention bias could mediate the relationship between ELS and depression. Based on previous evidence, we hypothesized that attention bias and the functional connectivity (FC) of attention networks might be the behavioral and neural markers of ELS. In addition, there were two possible mediation mechanisms which might elucidate the potential neural and cognitive mechanisms sustaining the relationship between ELS and depression.

## Method

### Participants

Dataset 1 contained 528 right-handed college students (157 males, age = 19.42 ± 1.40 years, age range: 16–26 years), 38 participants were excluded for its maximal motion between volumes in each direction > 3 mm, and rotation about each axis >3°. Dataset 2 contained 208 right-handed college students (86 males, age = 22.30 ± 1.47 years, age range: 18–27 years), 25 participants were excluded for its maximal motion between volumes in each direction >3 mm, and rotation about each axis >3° (see Table 1). Participants with a history of manic episodes, psychotic features or neurological illness were excluded. All participants were college students from Southwest University in China. They were informed of the procedure of the experiment, and finished written informed consent prior to participating in the study. For the participants under the age of 18 years, their parents were informed of the procedure and allowed them to participate in the experiment, and finished written informed consent prior to participating in the study. The study was approved by the Southwest University Brain Imaging Center Institutional Review Board. All research was performed in accordance with the relevant guidelines and regulations.

**Table 1:** Demographic data.

Variables	Dataset 1 (n = 528)			Dataset 2 (n = 208)		
	Mean	SD	Range	Mean	SD	Range
Age	19.42	1.40	16–26	22.30	1.47	18–27
ELS	37.35	8.34	25–72	35.39	9.16	25–77
Depression	7.05	6.45	0–30	7.24	5.76	0–27
Present stress	46.70	20.88	0–132	46.43	31.37	0-131

Note: SD = standard deviation;

### Measure of the behavior variable

#### The childhood trauma questionnaire

The Childhood Trauma Questionnaire-Short Form (CTQ-SF) was used to evaluate individual’s traumatic experience during childhood^41^. The CTQ consisted of 25 clinical questions and 3 validity items. All the items were rated on a 5-point Likert scale (ranging from 1 never true to 5 very often true) enquiring about 5 types of childhood trauma including emotional, physical and sexual abuse and emotional and physical neglect. The CTQ-SF has demonstrated good reliability and validity^42^. The Cronbach’s alpha of DAQ in the dataset 1 was 0.74, indicating that the present questionnaire had good construct validity.

#### Depression

Individual’s difference in depression was evaluated by Beck Depression Inventory^43^. The BDI-II is a 21-item self-report questionnaire which measured the severity of depressive symptoms within the past week. Each of the 21 items was rated on 4-point Likert-type scale ranging from 0–3. Participants who get higher score in the BDI tend to experienced more depressive symptoms. The BDI- II is a reliable measure which is widely used to assesses the severity of depressive symptoms from non-clinical to clinical samples^44^.The Cronbach’s alpha of the scale was 0.85 in this sample,

#### Current stress

The Current stress was accessed by the Adolescent Self-Rating Life Events Checklist^45^. It consisted of 27 common negative life events which belonged to multiple social-stress domains: family (e.g., “Physical punishment by parents”), school (e.g., “Failure in a test”), interpersonal (e.g., “Break up with close friends”), and personal physical diseases (see Table 1), these life events were suggested to be the most frequently occurred to Chinese adolescents^45^. Each item begins with whether the participants experienced the particular event, and then there is a five-point Likert scale (ranging from 1 “not at all” to 5 “extremely severe”) to evaluate the perceived stress of each life event. The Cronbach’s alpha of the scale was 0.95 in this sample,

#### Dot probe task

102 participants completed the experiment outside of the MRI scanner. Eprime 2.0 software (https://pstnet.com/products/e-prime/) was used to control the presentation of stimuli and to record response accuracy and response time. The dot probe task was designed according to MacLeod^46^. At the beginning of the task, there was a fixation cross presented for 500 ms on the screen, followed by a pair of faces for 500 ms, after which the dots appeared, then the dots disappeared. The participants were then asked to identify whether the small dots were arranged horizontally or vertically by pressing the associated key. Before the real experiment started, participants were given five practice trials to familiarize themselves with the procedure. The task contained 8 conditions: 4 emotion face pairs (neutral and happy, NH, neutral and sad, NS, neutral and anger, NF, neutral and fear) x 2 probe locations (in the location of the emotional face or the neutral face) and was comprised of 2 blocks of 151 trails (NH 40 trails, NS 37 trails, NA 37 trails, NF 37 trails), with a short break between the blocks. Attention bias scores were calculated as follows:

Attentional bias score = 1/2[(RpLe - RpRe) + (LpRe - LpLe)]^47^

R = right position; L = left position; p = probe; e = emotional face. In this equation, RpLe represents the mean reaction time when the probe presents in the left and the emotional face presents in the right, and so on. This equation estimates the “attention capturing” quality of emotional faces by subtracting the mean reaction times for probes presenting in the same position of the emotional face from the mean reaction times for probes presenting in the opposite position of the emotional face^47^. Positive bias scores represent the allocation of attention toward the emotional face compare with the matched neutral faces, and negative bias scores represent the allocation of attention away from the emotional face relative to the matched neutral faces, that is attention avoidance.

### RsfMRI data acquisition and preprocessing

#### Image acquisition

Whole-brain images were quarried by a Siemens 3.0 T magnetic resonance scanner. Participant’s high-resolution anatomical image data was acquired through a T1- weighted magnetization-prepared rapid gradient-echo (3d MP-RAGE) sequence in sagittal plane (inversion time = 900 ms, repetition time = 1900 ms, echo time = 2.52 ms, flip angle = 9°, resolution matrix = 256 × 256, thickness = 1.0 mm, slices = 176, voxel size = 1 × 1 × 1 mm^3^). The functional image was coregistrated to T1 image.

The resting-state fMRI data scanning took 8 minutes, all participants were instructed to have a rest while keeping eyes closed, but stay awake during scanning. Participant’s whole brain functional slices were acquired in descending order by gradient-echoplanar imaging (EPI) sequences, and the detailed parameters were as follows: repetition time (TR) = 2000, echo time (TE) = 30 ms, slices = 32, flip angle = 90°, field of view (FOV) = 220 mm × 220 mm, andvoxel size 3.4 × 3.4 × 4 mm^3^, thickness = 3 mm¸ slice gap = 1 mm.

#### Image data preprocessing

Imaging data was applied with SPM8 toolbox (http://www.fil.ion.ucl.ac.uk/spm/ software/spm8/) and Data Processing Assistant for Resting- State fMRI (DPARSF) based on MATLAB 2012a platform (MathWorks Inc., Natick, MA) to preprocess. The first 10 EPI images were removed considering that the participants need time to get used to the scanning circumstance, and slice timing were conducted in the remaining images. Then the neuroimaging data underwent standard realignment and normalization (MNI space). Next the fMRI time-series were entered into spatial smoothing (8 mm FWHM). Additionally, band-pass temporal filtering (0.01–0.1 Hz) were conducted and then remove nuisance signal including white matter (WM) and cerebrospinal fluid (CSF), global mean signal regression, and 6 motion parameters. Finally, data quality control was performed and removed the participants whose maximal motion in each direction>3 mm, and rotation>3°.

### Data analysis

#### Calculating of network measures

After preprocessing of resting-state MRI data, the power 264 template was adopted to extract the time course of each ROI^48^. Then 10 well-defined brain networks (contain 227 nodes) were chosen to enter into the follow-up analysis, including the default mode network (DMN), salience network (SN), fronto-parietal network (FPN), cingulo-opercular network (CON), sensorimotor network (SMN), visual network (VN), ventral attention network (VAN), dorsal attention network (DAN), auditory network (AN) and subcortical network (SUB). The within-network connectivity was calculated as follows:

$${W}_{a}\,=\,\frac{{\sum }_{{\rm{i}},{\rm{j}}\in {\rm{a}}}\,{A}_{i,j}\,}{{N}_{a}^{2}}$$

For each brain network (a∈1,2, ⋯,10), the within-network connectivity was calculated as the average connectivity across all the links of the network normalized by the square of the number of nodes (ROIs). 𝐴𝑖𝑗 represent the 227 × 227 connectivity matrix; 𝑁𝑎 is the number of nodes within network 𝑎; 𝑖 and 𝑗 notes the Power ROIs. Higher level of within-network connectivity indicated stronger interaction between those regions within the particular network.

The pairwise connectivity between networks was computed as follows:

$$P{B}_{a-b}=\frac{{\sum }_{{\rm{i}}\in {\rm{a}},{\rm{j}}\in {\rm{b}}}\,{A}_{i,j}}{{N}_{a}{N}_{b}}$$

The pairwise connectivity between networks (𝑃𝐵𝑎−𝑏) was computed as the average connectivity across all the links between two networks, 𝑎 and 𝑏 (𝑎, 𝑏∈1,2, ⋯,10; 𝑎 ≠ 𝑏), normalized by the product of the number of nodes within the two networks^49^. Higher level of between-network connectivity indicated stronger interaction between those regions of the two networks.

#### Brain–behavior correlation analyses

We conducted pearson correlations analysis between the within/between-networks of connectivity and ELS, controlling for age and sex. The results were corrected by Benjamini and Hochberg FDR (BH_FDR) correction^50^.

#### Prediction analysis

To test the robustness of the brain-behavior relationship, we performed a machine-learning method named linear support vector regression (SVR) and cross-validation procedure with balanced ten-fold^51^. ELS was taken as the dependent variable and the network connectivity were taken as independent variables in the linear regression algorithm. The r_(predicted, observed)_ was estimated by a four-fold cross-validation, and represent the prediction accuracy of the independent variable. We divided the data into four folds in order to keep the distributions of independent and dependent variables balanced. The prediction model was trained on the 75% sample and then was tested on the remaining participants. This procedure was conducted for 4 times to acquire a final r_(predicted, observed)_ representing the correlation between the predict value and the actual value.

#### Generalization to a different sample

To examine to what extent the results could generalize to other sample, the same analysis was applied to Dataset 2. The analysis process was as follows: 1), preprocess the resting-state fMRI data, 2), construct brain networks based on the power 264 template, 3), calculate the within/between network connectivity, 4), Person correlation analysis was performed to investigate the relationship between ELS and network measures.

#### Mediation analysis

To examine whether the within-network connectivity of the VAN could explain the relationship between ELS and depression, a mediation analysis was conducted. The mediation model could partly explain the causal pathway by which the dependent variable (Y) was affected by the independent variable (X) affects a dependent variable (Y). Mediation analyses were performed by applying the indirect macro designed for SPSS^52^. In the current study, X is the ELS, Y is the depression, and M is the within-network connectivity of the VAN. Age and sex were entered as covariates in the mediation model. This macro uses bootstrapped sampling to estimate the indirect mediation effect. In this alogorithm, 2,000 bootstrapped samples were drawn, and bias corrected 95% bootstrap confidence intervals (CI) were reported. CI that do not include zero indicate a significant indirect effect of the independent variable on the dependent variable through the mediators. Besides, the same mediating method was performed to explore whether attention bias of a sad face could explain the association between ELS and depression.

## Results

### The neural correlates of ELS

**Figure 1:**
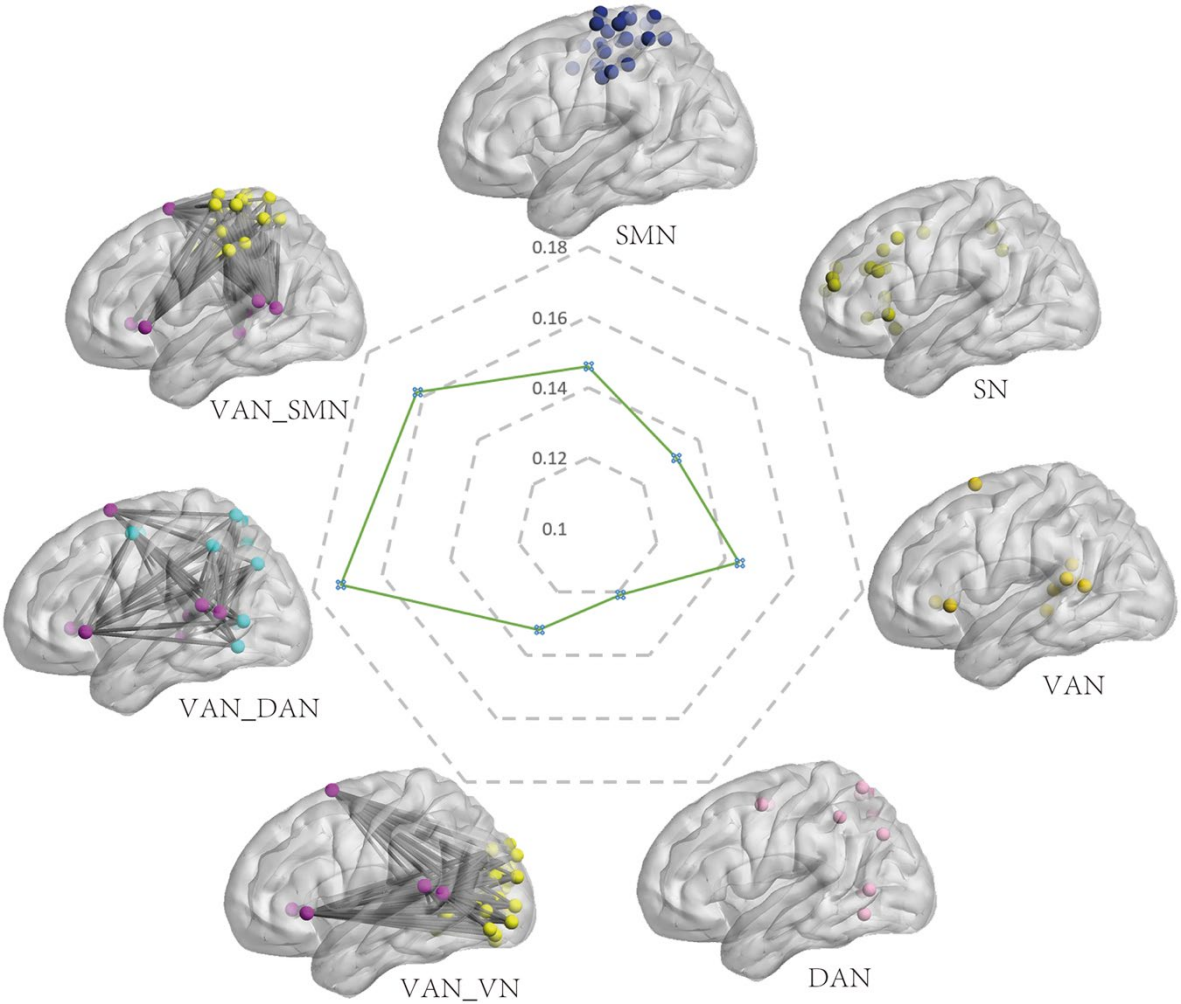
Within/between-network connectivity associated with ELS. The radar map shows the correlation coefficients of the within/between-network connectivity with ELS. Abbreviations: VAN, ventral attention network, DAN, dorsal attention network, SN, salience network, SMN, sensorimotor network, VN, visual network.

**Figure 2:**
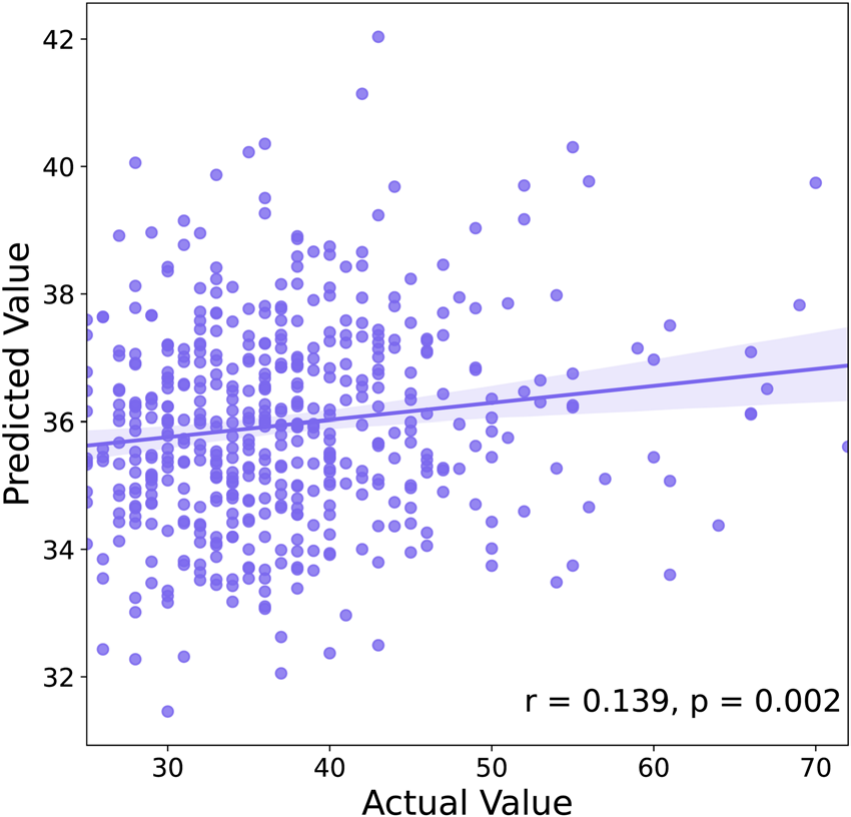
The prediction results. The within-network connectivity of VAN, DAN, SN, SMN and the between-network connectivity of VAN_DAN, VAN_SN, VAN_SMN could predict individual difference of ELS.

The demographic data are presented in Table 1. There was no gender difference in ELS (t = 2.682, p = 0.377), and ELS was not related to age (r = 0.033, p = 0.439). Within-network results indicated that ELS is positively correlated with within-network connectivity of the VAN, DAN, SMN-H, SN, and between-network of the VAN-DAN, VAN-VN, VAN-SMN (see Table 2 and Figure 1). Then prediction analysis was performed to test the robustness of the relationship between ELS and the within-network connectivity of these networks. Prediction results demonstrated that within/between-network connectivity of these networks could predict ELS (r = 0.139, p < 0.005, see Figure 2), after controlling for age, gender and current stress. Due to the relatively small ample size of the Dataset 2 and its resultant low statistical power, the Dataset 2 only partly verified the present results. The results of Dataset 2 indicated that ELS was positively correlated with BDI score (r = 0.230, p < 0.005), the within-network connectivity of the VAN (r = 0.224, p < 0.005) and the DAN (r = 0.165, p < 0.05).

**Table 2:** Within/between-network connectivity associated with ELS.

	Network	r	p	^p^B H_FDR
Within-network	VAN	0.144	0.001	<0.05
	DAN	0.121	0.007	<0.05
	SN	0.132	0.003	<0.05
	SMN	0.146	0.001	<0.05
Between-network	VAN-DAN	0.172	0.001	<0.05
	VAN-SMN	0.162	0.001	<0.05
	VAN-VN	0.132	0.001	<0.05

Abbreviations: VAN, ventral attention network, DAN, dorsal attention network, SN, salience network, SMN, sensorimotor network, VN, visual network.

### Correlations between ELS, network measures and depression

A correlation analysis was conducted to explore the relationship between ELS, network measures and depression. After controlling for age, gender and current stress, ELS was significantly correlated with depression (r = 0.204, p < 0.001), and the within-network connectivity of the VAN was also associated with depression (r = 0.137, p < 0.005). These results suggested that a intensely association exists between the within-network connectivity of the VAN, ELS and depression. To test the hypothesis that attention bias sustains the relationship between ELS and depression, we also investigate the relationship between ELS, attention bias and depression.

### Mediation results

**Figure 3:**
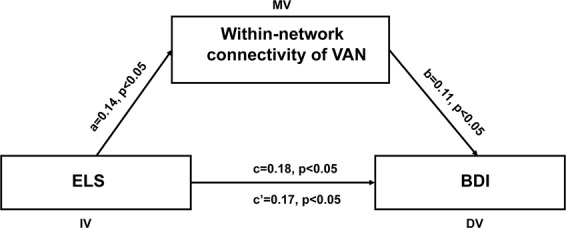
The within-network connectivity of VAN (Ventral attention network) mediate the relationship between ELS (Early life stress) and depression. The depicted diagram shows that ELS predicts displaced depression through the within-network connectivity of VAN after controlling for age, gender. Standardized regression coefficients are present in the path diagram.

**Figure 4:**
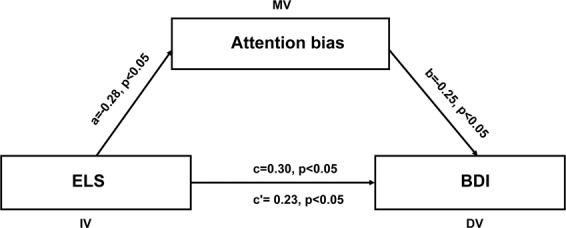
Attention bias mediate the relationship between ELS (Early life stress) and depression. The depicted diagram shows that ELS predicts displaced depression through attention bias after controlling for age, gender. Standardized regression coefficients are present in the path diagram.

The first mediation analysis indicated that the within-network connectivity of the VAN significantly mediate the relationship between ELS and depression (indirect effect = 0.01, 95% CI = [0.004, 0.04], p < 0.05, see Figure 3), Standardized regression coefficients are present in the path diagram, which represent the covariant relationship between two variables. The second mediation analysis indicated that the attention bias to a sad face had a significant mediating effect on the relationship between ELS and depression (indirect effect = 0.07, 95% CI = [0.007, 0.125], p < 0.05, see Figure 4)

## Discussion

This study extends existing knowledge about the neural basis of ELS by revealing that FC within/between large-scale brain networks were related to the individual differences of ELS. Fist, positive associations were observed between ELS and the within-network connectivity of the VAN, DAN, SN, SMN. Second, we found a positive relationship between ELS and the between-network connectivity of the VAN- DAN, VAN-VN, VAN-SMN. Finally, we found that attention bias and the within-network connectivity of the VAN mediate the relationship between ELS and depression. These results therefore provide a convincible neurobiological association for the changes in attention associated with childhood trauma and depression.

Positive associations were observed between ELS and the within-network connectivity of the VAN, DAN and the between-network connectivity of the VAN-DAN, which fitted well with previous studies that aberrant function of brain regions in attention network were associated with ELS and depression. For example, exposure to ELS was associated with increased activation of inferior parietal lobule during working memory task^53^. Previous studies detected increased functional connectivity between dmpfc, dlpfc (key node of DAN), vmpfc and ACC^54^ in MDD patients. The VAN was confirmed to be involved in stimulus-driven attention^29^. For instance, detecting a salient target might require the involvement of the VAN (i.e., “invalid” targets in the Posner spatial cueing paradigm)^55–58^. Whereas the DAN embodies top-down attention control^59,60^. DAN activity might increase when individuals orientate their attention to the current task^56,61,62^

Generally, these two networks tend to cooperate during normal cognitive processes. During an ongoing cognitive process, that task-relevant signals from the dorsal attention network might “filter” stimulus-driven signals in the ventral attention network, while stimulus-driven “circuit-breaking” signals from the ventral attention network offer an interruption to the dorsal attention network, reorienting the attention toward salient stimuli^63^. Several studies have demonstrated the association between childhood trauma and aberrant threat detection and response. Specifically, clinical study suggested that vigilance mediates the association between childhood neglect and negative symptoms in male psychotic patients^64^, exposure to ELS is associated with attention bias to a threatening stimulus, such as a fearful face^65^, or the emotional valence word^66–68^. Overall, this increased within/between-network connectivity of attention systems might indicate a higher state of vigilance and awareness in individuals exposed to ELS.

In addition, the results also demonstrated that ELS was positively correlated with the within-network connectivity of the SN, SMN, and the between-network connectivity of VAN-VN, VAN-SMN. The SN is suggested to be involved in responding to behaviorally salient stimuli^69^ and implement the coordination of behavioral responses^70^. Increased SN activity has been observed during situations in which changing behavior is more likely to be needed^71^. Previous study has indicated that the SN is involved in supporting stable attention for a current goal^72^. In this study, this association between ELS and the within-network connectivity of the SN might suggest constant attention toward the external environment. The abnormal anatomical structure of the SMN is widely reported in ELS related studies^73,74^. The SMN is related to the processing of incoming sensory stimuli and generating motor outputs^75–79^. Previous studies have suggested that depressive temperament was associated with alternated neuronal variability in the SMN^80^, Increased within-network connectivity of the SMN might support being more reactive to internal and external signals for initiating response. The VN was involved in processing primary visual information and projecting to higher sensory areas^81^. The stronger interaction between the SMN, VN and VAN might indicate a more sensitive perception of internal and external stimulus.

Further analysis revealed that there were positive association between the within-network connectivity of the VAN, attentional bias and ELS, which might suggest that adults who have experienced ELS have an altered within-network connectivity of the VAN associated with attention bias. In order to explore the neural and cognitive mechanisms for the influence of ELS on depression, we constructed two mediation models to decipher the potential relationship between ELS, depression, within-network connectivity of the VAN and attention bias. There is no doubt that attentional bias is a key cognitive feature in people with ELS, but the orientation of attentional bias is still controversial. Some studies indicated that ELS is related to attentional bias toward threatening or sad face^82,83^, while others suggested attentional bias away from threatening stimuli^84,85^. The present results supported the latter point. According to Posner’s study, longer stimulus onset asynchronies (more than 300 ms) might result in a reliable disadvantage for valid trails, during which the attention was first engaged by the cue and then disengaged from it (that is attention avoidance). Presumably, attention avoidance might be a defensive response to reducing exposure to negative stimuli, but from a long-term perspective it attentional avoidance might hinder effective emotion regulation^86,87^. Specifically, exposure to ELS might lead to attentional avoidance, which might associate with ineffective emotion regulation. Eventually, individual who has experienced ELS will develop a higher depressive state after stress events. Previous studies demonstrated that major depressive disorder was associated with abnormal connectivity of regions involved in cognitive control of attention^88,89^, indicating an aberrant attention process to salient cues. As for the role of the VAN in the relationship between ELS and depression, one possible explanation is that increased within-network connectivity of VAN might relate to a stronger stimulus-driven attention toward threaten stimuli, which conform to the sensibility to threatening stimuli in individual with higher depression.

Altogether, the present study provided behavioral and neural evidence for the alternations of ELS on individual’s attention. Based on these results, we can infer that attention is a key factor to explain how individual who have experienced ELS develop depression. Besides, attention bias modification training could serve as a potential intervention method to reduce the negative impact of ELS.
